# Biliary dilation using a screw-type device for endoscopic ultrasound-hepaticogastrostomy in a relapsed primary sclerosing cholangitis case post-liver transplantation

**DOI:** 10.1055/a-2638-5381

**Published:** 2025-07-15

**Authors:** Mitsuru Okuno, Fumiya Kataoka, Hiroshi Araki, Eiichi Tomita, Hidetoshi Matsunami, Hisataka Moriwaki, Masahito Shimizu

**Affiliations:** 1 73505 Department of Gastroenterology, Matsunami General Hospital, Gifu, Japan; 2 73505 Department of Surgery, Matsunami General Hospital, Gifu, Japan; 3 476117 First Department of Internal Medicine, Gifu University Hospital, Gifu, Japan

Primary sclerosing cholangitis (PSC) can cause biliary strictures and cirrhosis. Although liver transplantation is curative for decompensated cirrhosis, PSC can relapse posttransplantation 1 2 . Even though balloon dilatation is effective for biliary strictures 3 , endoscopic retrograde cholangiopancreatography (ERCP) is sometimes challenging due to surgical reconstruction. Alternative approaches include percutaneous transhepatic biliary drainage (PTBD) and endoscopic ultrasound (EUS)-guided biliary drainage (EUS-BD).

A 49-year-old male patient underwent liver transplantation with choledochojejunostomy due to PSC. Liver cirrhosis after liver transplantation was diagnosed at 59 years old. He visited our hospital with abdominal pain and was diagnosed with cholangitis. ERCP using the enteroscopy was attempted, but the endoscope failed to reach the biliary anastomosis. The PTBD tube was placed for continuous biliary drainage, and cholangiography indicated PSC relapse. After 6 months, the PTBD tube dislodged, prompting us to plan an EUS-hepaticogastrostomy (EUS-HGS) for biliary drainage to improve the patient’s quality of life. A thick bile duct wall was detected using EUS and punctured using a 22-G needle (EZ Shot 3 Plus; Olympus Medical Systems, Tokyo, Japan). A 0.018-in. guidewire (Fielder 18; Olympus) was inserted in the intrahepatic bile duct (IHBD). Due to the thick and hardened IHBD, a 7-Fr screw (Tornus ES; Olympus) was used for biliary dilation. A 7-Fr plastic stent was placed after dilation ( Fig. 1 ). The IHBD and anastomosis were dilated 1 week later using a 6-mm balloon catheter (REN; Kaneka, Tokyo, Japan) via the EUS-HGS route. Liver enzyme levels improved, and a plastic stent was placed to maintain bile flow ( Fig. 2 , Video 1 ).

**Fig. 1:**
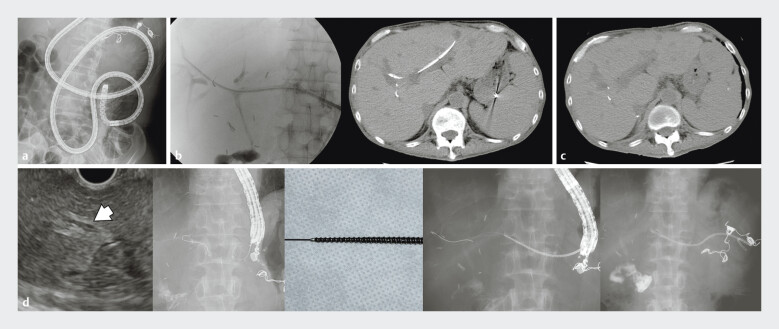
**a** Endoscopic retrograde cholangiopancreatography performed to evaluate the bile duct and place biliary drainage using an enteroscope; however, the scope could not reach the biliary anastomosis. **b** Percutaneous transhepatic biliary drainage (PTBD) performed as an alternative, with primary sclerosing cholangitis relapse indicated based on cholangiogram. PTBD tube placed for continuous biliary drainage. **c** PTBD tube accidentally dislodged 6 months later. Computed tomography reveals the dilated intrahepatic bile duct. **d** Endoscopic ultrasound-guided hepaticogastrostomy performed using 22-G needle (EZ Shot 3 Plus; Olympus Medical Systems, Tokyo, Japan). A 0.018-in. guidewire (Fielder 18; Olympus) is carefully inserted into intrahepatic bile duct (IHBD). Due to thickened and hard IHBD, the screw shape 7-Fr dilater (Tornus ES; Olympus) is used for biliary dilation. After successful dilation, a 7-Fr plastic stent is placed. ERCP: endoscopic retrograde cholangiopancreatography; PTBD: percutaneous transhepatic biliary drainage; PSC: primary sclerosing cholangitis; CT: computed tomography; EUS-HGS: endoscopic ultrasound-guided hepaticogastrostomy; IHBD: intrahepatic bile duct.

**Fig. 2:**
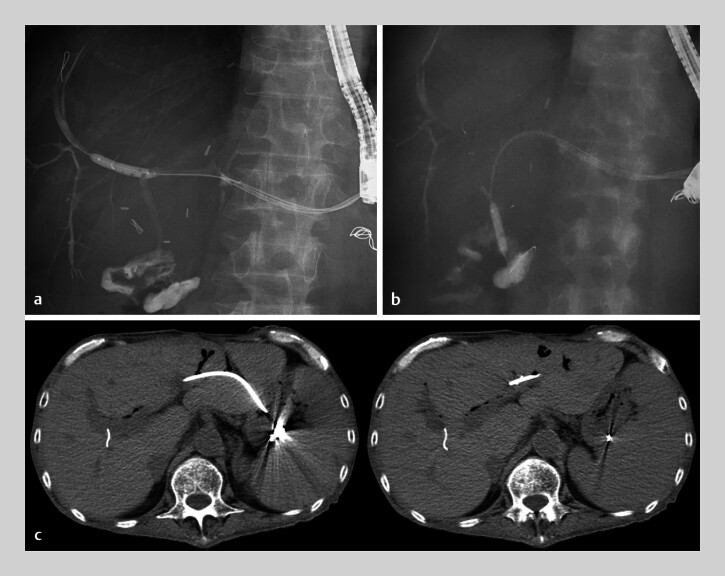
Intrahepatic bile duct (IHBD) ( **a** ) and biliary anastomosis ( **b** ) are further dilated using a 6-mm balloon catheter (REN; Kaneka, Tokyo, Japan) via endoscopic ultrasound-guided hepaticogastrostomy route. After balloon dilation, computed tomography reveals a dilated IHBD, with improved gastrointestinal tract access ( **c** ). IHBD, intrahepatic bile duct; EUS-HGS, endoscopic ultrasound-guided hepaticogastrostomy; CT, computed tomography.

Endoscopic ultrasound-guided hepaticogastrostomy (EUS-HGS) in a case of suspected primary sclerosing cholangitis relapse after liver transplantation. A thick bile duct wall detected using endoscopic ultrasound, with a screw-shaped 7-Fr dilator (Tornus ES; Olympus) used for biliary dilation. After stent placement, further dilation of the bile duct and anastomosis was performed using a 6-mm balloon catheter via the EUS-HGS route. EUS-HGS, endoscopic ultrasound-guided hepaticogastrostomy; PSC, primary sclerosing cholangitis; EUS, endoscopic ultrasound; IHBD, intrahepatic bile duct.Video 1

In this case, PSC relapse and long-term PTBD placement thickened and hardened the bile duct wall, complicating EUS-HGS. Tornus ES successfully accessed the bile duct through gradual insertion via screw rotation rather than axial guidewire advancement. Hence, even with a fine guidewire, a screw-shaped dilator can prevent unstable scope movement by pushing against the bile duct wall or splashing out of the guidewire. Considering the difficulty of EUS-BD in liver transplantation cases owing to bile duct stiffness, the screw-type dilator provides a valuable option for EUS-BD procedures.

Endoscopy_UCTN_Code_TTT_1AS_2AI

## Notes

Endoscopy E-Videos https://eref.thieme.de/e-videos
*E-Videos* is an open access online section of the journal *Endoscopy* , reporting on interesting cases and new techniques in gastroenterological endoscopy. All papers include a high-quality video and are published with a Creative Commons CC-BY license. Endoscopy E-Videos qualify for HINARI discounts and waivers and eligibility is automatically checked during the submission process. We grant 100% waivers to articles whose corresponding authors are based in Group A countries and 50% waivers to those who are based in Group B countries as classified by Research4Life (see: https://www.research4life.org/access/eligibility/ ). This section has its own submission website at https://mc.manuscriptcentral.com/e-videos .
